# New Appliances and Things Medical

**Published:** 1905-01-28

**Authors:** 


### NEW APPLIANCES AND THINGS MEDICAL.

[We shall be glad to receive at our Office, 28 & 29 Southampton Street, Strand, London, W.C., from the manufacturers, specimens of all new preparations and appliances which may be brought out from time to time.]

### HUGON'S REFINED BEEF SUET, ATORA BRAND.

(HUGON AND CO., PENDLETON, MANCHESTER.)

THE preparation before us is a concentrated beef suet, and consists practically of pure beef fat. One of the most important factors in the digestibility of fat is its melting point, and in this respect the most digestible fat we have is goose fat, which possesses a very low melting point. Another factor of great importance is the extent to which the fat lends itself to minute mechanical division. In the preparation of suet puddings and pastry, this question of the disintegration of the admixed fat is of very great importance, a so-called short pastry being less likely to cause trouble in digestion than a "flaky" pastry. The preparation before us lends itself well to minute disintegration, and in this way makes relatively light and digestible suet puddings. It keeps well, and may be used along with butter or lard for making pastry. The preparation is to be recommended.

### BANANA PREPARATIONS.

(THE BANANA FOOD COMPANY, 46 ARTILLERY LANE, BISHOPSGATE, LONDON, E.C).

THE extraordinary productiveness of the banana tree long since struck many travellers in tropical and sub-tropical regions. Amongst others Humboldt drew attention to the smallness of the area required to support a population if planted with bananas. Bananas seem for a considerable time to have been a source of arrowroot, but have only relatively recently been introduced systematically as a source of ordinary food. The fruit itself, as is well known, consists of a skin and a fruit substance, and it is interesting to note that of the whole fruit no less than 40 per cent. is composed of skin, or almost half by weight. The essential factor in the food preparations before us is banana flour; this is prepared from the dried unripe fruit. The unripe banana flour differs from the contents of the ripe fruit very markedly in its proportion of starch and sugar, the flour containing 77·67 per cent. of starch, the ripe fruit only 1·85 per cent. Speaking generally, banana flour is rich in carbohydrates and relatively poor in proteids. In Bananina no attempt has been made to increase appreciably the proteid content, and it is directly stated that nothing but bananas are used in its preparation. The food may be made with milk or water as directed upon the tin. The banana bread is quite palatable, and the taste of bananas is not very evident. The idea of combining banana flour, relatively poor in proteids, with oatmeal, this latter being distinguished by its relatively high proteid content, is a good one, and the result should have a large sphere of usefulness.

### PHILLIPS' MILK OF MAGNESIA.

(PHILLIPS CHEMICAL CO., NEW YORK AND LONDON).

GASTRIC hyperacidity is usually treated by attempting to neutralise the acid by an alkali. For this purpose various alkalies are used; perhaps the most common one is bicarbonate of soda, others having also a specific action upon other factors of importance in many forms of dyspepsia. The disadvantage of the carbonates, however, is that neutralisation of them involves the evolution of $CO_2$ gas, and therefore tends at first to increase rather than diminish gastric distention. This evil is prevented by the use of the alkaline phosphates, and the hydrates. These latter bodies, with the almost single exception of the magnesium hydrate are too caustic and irritating for use. The preparation before us consists of the hydrate of magnesium, one fluid ounce containing 24 grains of $Mg(OH)_2$. This preparation should be useful and certainly is scientifically conceived; it may, however, be parenthetically remarked that the official (B.P.) carbonate of magnesium contains as an impurity 10 per cent. of $Mg(OH)_2$. The milk of magnesia is practically free from carbonate.

